# Identification of RALA as a Therapeutic Target and Prognostic Predictor of Osteosarcoma

**DOI:** 10.1155/2023/1150768

**Published:** 2023-02-07

**Authors:** Gentao Fan, Yan Zhu, Hao Zhu, Lingfeng Yu, Zhen Wang, Chenjun Zhai, Guangxin Zhou, Jianning Zhao, Yicun Wang

**Affiliations:** ^1^Jinling Hospital Department of Orthopaedics Nanjing Medical University, Nanjing, Jiangsu 210002, China; ^2^Department of Orthopedics, Affiliated Jianhu Hospital of Nantong University, No. 666 Nanhuan Road, Jianhu, Yancheng, 224700 Jiangsu, China; ^3^Department of Orthopaedics, Affiliated Jinling Hospital Medical School of Nanjing University, Nanjing, Jiangsu 210002, China; ^4^Department of Orthopedics, Yixing People's Hospital, 75, Tongzhenguan Road, Yixing 214200, China

## Abstract

**Background:**

Osteosarcoma (OS) is the most common primary aggressive sarcoma of bone, with massive aberrant expression of oncogenes related to the development of OS. RALA, a kind of small Ras-like guanosine triphosphatases, has been identified as a potential therapeutic target in several types of tumor, but its role in OS remains largely unknown.

**Methods:**

Abnormal expression of RALA was proven in the Cancer Genome Atlas (TCGA), Genotype-Tissue Expression (GTEx), Therapeutically Applicable Research to Generate Effective Treatments (TARGET), and RNA-sequence of samples and cell lines. The role of RALA in OS was analyzed in terms of DNA methylation, immune cell infiltration, and patient survival. The cancer-promoting effect of RALA was demonstrated in cell lines and xenograft osteosarcoma models. A prognostic scoring model incorporating RALA as an indicator was established with the clinical samples that we collected.

**Results:**

The results showed that RALA was highly expressed in human OS tissues and cell lines. Survival analysis demonstrated that RALA was the sole independent risk factor for poor overall survival and disease-free survival in OS patients and impacted the proportion of infiltrating immune cells and DNA methylation in the OS tumor microenvironment. By gene-gene interaction analysis, we found that the expression of RALA was highly correlated to the expression of ABCE1. Similar to RALA, upregulated ABCE1 is correlated with poor survival outcome of OS patients. In addition, the functional experiment demonstrated that higher expression of RALA promoted the proliferation, migration, and invasion of OS cells. In vivo results were similar with the in vitro results. We examined m6a methylation-related genes and found that m6A methylation is responsible for the abnormal expression of RALA. Finally, the prognostic prediction model of RALA could be used to predict the long-term outcome of OS patients.

**Conclusions:**

We identified RALA as an oncogene in OS, and RALA upregulation in a concerted manner with ABCE1 was significantly associated with worse outcomes of OS patients. Targeting RALA may prove to be a novel target for OS immunotherapy in future clinical practice.

## 1. Introduction

Osteosarcoma (OS) is the most common primary malignant tumor of bone occurring most frequently in adolescence, with a combined global incidence of 3-4 cases per million people [1]. OS consists of malignant mesenchymal osteoblasts producing immature bone and osteoid tissues. The disease progresses rapidly and is locally aggressive and easy to metastasize, resulting in poor prognosis [2]. Despite current standard treatment, nearly one-third of OS patients experienced disease recurrence or metastasis, and about 20% patients had presented pulmonary metastasis at the time of diagnosis [3], when the 5-year survival rate is only about 20%, and the median survival is less than 8 months [4]. At the same time, there is no widely effective targeted therapy for patients with metastatic and recurrent OS. It is therefore necessary to gain a better understanding of the molecular mechanism underlying OS.

Ras-like guanosine triphosphatases (Ral GTPases) are members of the Ras superfamily of small GTPases [5]. Previous studies indicated that Ral GTPases harbor activating mutations in about 25% of all human cancers, making them the most frequently mutated oncogenes [6]. Various physiological functions have been associated with Ral GTPases, including neuronal plasticity, immune response, and glucose and lipid homeostasis [7]. Ral GTPases include RALA and RALB. Numerous clinical and experimental studies have reported that RALA is a significant driver of diverse biological processes like proliferation, migration, and invasion of various human cancers, including skin, lung, pancreatic, colon, prostate, and bladder cancers [8]. Several preliminary studies suggested that RALA can participate in immune cell differentiation and the regulation of related processes [7, 9]. Low expression of RALA affects the recognition and migration of NK cells, reduces the toxicity of NK cells, interferes with the maintenance of NK cell homeostasis, and inhibits the immune surveillance function of NK cells [10]. The role of RALA in the immune microenvironment in OS is not clear. The aim of present study was to explore new methods of identifying targets in OS and clarify the role of RALA in the biological process of OS from multiple dimensions in vivo and in vitro by using bioinformatic methods in combination with molecular biology experiments for the sake of providing new ideas for the treatment of OS.

## 2. Materials and Methods

### 2.1. Cell Line and Culture

The human osteoblast cell line hFOB1.19 and human osteosarcoma cell lines (U2OS, 143B, and MG63) obtained from the Cell Bank of Type Culture Collection of the Chinese Academy of Sciences (Shanghai, China) were cultured in DMEM supplemented with 10% fetal bovine serum (FBS; Gibco, Thermo Fisher Scientific, Inc.) and maintained at 37°C in a humidified atmosphere containing 5% CO_2_.

### 2.2. Patients and Tissues

Fresh human osteosarcoma tissue samples and normal tissues were obtained from patients undergoing surgical procedures at Jinling Hospital (Nanjing, China) between 2007.01 and 2021.12. Normal bone or connective tissues that were within 2 cm from the edge of osteosarcoma tissue were collected as the cancer adjacent normal tissues. All patients in the study, or their guardians, provided written consent prior to participation. The study protocol was approved by the Ethics Committee of the said hospital (Nanjing, China).

### 2.3. Public Data

The raw public data were downloaded from the Cancer Genome Atlas (TCGA, https://portal.gdc.cancer.gov/) dataset, Genotype-Tissue Expression (GTEx, https://gtexportal.org/) dataset, Therapeutically Applicable Research To Generate Effective Treatments (TARGET, https://ocg.cancer.gov/programs/target) dataset, Tumor Immune Single-cell Hub (TISH, http://tisch.comp-genomics.org/), STRING (https://cn.string-db.org/), and the Timer (https://cistrome.shinyapp-s.io/timer/) dataset. Patient information for OS and sarcoma was downloaded from TARGET and TCGA. Information on normal tissues was downloaded from GTeX. Immunization-related information was downloaded from Timer. Single-cell sequence data were downloaded from TISH. Protein-protein data were downloaded from STRING.

### 2.4. Cell Transfection

Mammalian expression plasmids encoding the human RALA open reading frame (ORF) were purchased from GenScript (Shanghai, China). They were transfected into OS cells using Lipofectamine 3000^®^ reagent (Invitrogen; Thermo Fisher Scientific, Inc.), using an empty plasmid as control vector. Cells were harvested 24 h after transfection, or when the cell monolayer reached 100% confluence.

### 2.5. Immunohistochemistry and Evaluation

Standard immunohistochemical procedures were used. The sample was deparaffinized with xylene, rehydrated with ethanol, and incubated with 3% H2O2 for 5 min to block endogenous peroxidase activity. Then, antigen retrieval was performed by incubating the samples with sodium citrate buffer (pH 6.0) for 20 min at 95°C. After blocking with 5% normal goat serum for 10 min at 20°C, the sections were incubated with polyclonal antibodies against RALA (1 : 1000, Servicebio, Wuhan, China) and Ki67 (1 : 500, Servicebio, Wuhan, China). Then, a hematoxylin-eosin (H&E) staining kit (Solarbio, Beijing, China) was used for H&E staining. The images were captured with the Olympus FSX100 microscope (Olympus, Japan).

RALA expression level was evaluated by two pathologists independently. The intensity of staining was scored as follows: 0 (negative), 1 (weakly positive), 2 (moderately positive), and 3 (highly positive). The proportion of staining was scored according to the percentage of positively stained cells in the field as follows: 0 (0%), 1 (1-10%), 2 (11-40%), 3 (40-80%), and 4 (80%-100%). RALA immunopositivity scores were calculated by IHC as the sum of the intensity and extent scores. According to their RALA expression level, OS patients were classified into two groups: high-expression group (IHS≧4, *n* = 50) and low-expression group (HIS < 4, *n* = 50).

### 2.6. Western Blotting

Tissue samples were frozen in liquid nitrogen and ground into powder. Total protein was extracted using RIPA lysis buffer (Beyotime Institute of Biotechnology) supplemented with a protease and phosphatase inhibitor mixture (Thermo Fisher Scientific, Inc.) on ice for 30 min. Lysates were then centrifuged for 12 min (12,000 × g; 4°C), and the supernatant was collected. The protein concentration was measured using the Pierce BCA Protein Assay Kit (Thermo Fisher Scientific, Inc.), and proteins were separated via 10% SDS-PAGE. The separated proteins were subsequently transferred onto PVDF membranes (cat. no. 88518; Thermo Fisher Scientific, Inc.), which were then blocked at room temperature for 1 h using BSA blocker (cat. no. 37520; Thermo Fisher Scientific, Inc.). The membranes were then incubated with monoclonal anti-RALA (1 : 5000; cat. no. 3526; Cell Signaling Technology, Inc.) or anti-GAPDH (1 : 2000; cat. no. sc-25778; Santa Cruz Biotechnology, Inc.) primary antibodies for 24 h at 4°C. Following the primary antibody incubation, the membranes were incubated with secondary antibody (1 : 2000; cat. no. sc-2005; Santa Cruz Biotechnology, Inc.) at room temperature for 2 h. Protein bands were visualized with an enhanced chemiluminescence kit (cat. no. 34580; Thermo Fisher Scientific, Inc.), and densitometric analysis was performed using ImageJ software (version: x 64,1.48 U; NIH).

### 2.7. Reverse Transcription-Quantitative PCR (RT-qPCR)

Total RNA was extracted from cells and tissue samples for further study using TRIzol^®^ reagent (Invitrogen; Thermo Fisher Scientific, Inc.). The RNA concentration was measured using a microplate reader. To quantify RALA mRNA expression levels, 1 *μ*g total RNA was reverse transcribed into cDNA using avian myeloblastic virus reverse transcriptase and Oligo(dT) primers (both Takara Bio, Inc.). The reaction was incubated as follows: 16°C for 5 min, 42°C for 60 min, and 70°C for 10 min. Then, the RT-PCR mixture, consisting of specific primers for RALA and GAPDH and SYBR Green dye (Invitrogen; Thermo Fisher Scientific, Inc.), was incubated under the following conditions: 95°C for 5 min, followed by 40 cycles of 95°C for 30 s, 60°C for 30 s, and 72°C for 1 min. The following primer sequences were used for the RT-qPCR: RALA (forward: 5′-ATGGCTGCAAATAAGCCCAAG-3′ and reverse: 5′-TGTCTGC--TTTGGTAGGCTCATA-3′); GAPDH (forward: 5′-CGAGCCACATCGCTCAGAC-A-3′ and reverse: 5′-GTGGTGAAGACGCCAGTGGA-3′). Expression levels were quantified using the 2^−ΔΔCq^ method and normalized to GAPDH.

### 2.8. Cell Proliferation Assay

The proliferative ability of MG63 cells was analyzed using 5-ethynyl-2'-deoxyuridine (EdU) Cell Proliferation Assay kit (Takara Bio, Inc.) to incorporate 5-ethynyl-2'-deoxyuridine (EdU) according to the manufacturer's protocol. Data were analyzed using Image Pro Plus software (version: 7.0; Media Cybernetics, Inc.)

### 2.9. Cell Migration and Invasion Assay

The migratory ability of OS cells was detected using wound healing assay. The treated cells (100% confluence) were cultured in 6-well cell culture plates and scraped with the tip of 200 *μ*l pipette tips. Wound closure was photographed using a photomicroscope (BX51; Olympus Corporation) at 0 and 24 h after being incubated in serum-free medium.

The invasive ability of OS cells was analyzed using 12-well Millicell cell culture inserts (Merck KGaA). MG63 cells suspended in DMEM with 1% FBS were added to the upper chamber 24 h after transfection, DMEM supplemented with 10% FBS was plated into the chambers, and the Transwell plates were incubated for 20 h in a 5% CO_2_ atmosphere, which permitted MG63 cells to migrate and penetrate the gelatin-coated polycarbonate membranes towards the upper chamber containing more nutritious DMEM with a higher FBS content. The cells on the outboard surface of the inserts were washed, fixed (5% paraformaldehyde, Sangon Biotech; room temperature; protect from light), stained (0.2% purple crystal, Sangon Biotech), and visualized using a photomicroscope. (BX51; Olympus Corporation) (in five randomly selected fields of view).

### 2.10. Establishment of Tumor Xenografts in Mice

Male SCID mice (nu/nu; age, 6 weeks; weight, 18 g) were purchased from the Model Animal Research Center (Nanjing, China) and housed under specific pathogen-free conditions (28°C; humidity, 50%; light time, 10 hours; conventional feed) in Nanjing University. MG63 cells transfected with RALA overexpression plasmid were subcutaneously injected into 6-week-old SCID mice (2 × 10^6^ cells/mouse, six mice/group). The mice were sacrificed 24 days after injection with CO_2_ (17.5% cage volume/min for 5 min), and the formed tumors were removed. Tumor volumes were calculated using the following formula: tumor volume = [length × (width)^2^]/2. A portion of the tumor tissue was fixed in 4% paraformaldehyde for 24 h at 4°C and further processed for hematoxylin and eosin (H&E) and IHC staining for RALA and Ki67 expression. The remaining tissue was used for protein extraction. These experiments were repeated three times. The animal experimental protocol was approved by the Institutional Review Board of Jinling Hospital and performed in accordance with the Guide for the Care and Use of Laboratory Animals (National Institutes of Health). The ethics approval was obtained in September 2019.

### 2.11. Statistical Analysis and Visualization

Data from Western blotting, EdU, migration, invasion, and wound healing assays, together with the animal experiments, were representative of at least three independent experiments. All sample data were analyzed using SPSS software (version 11.0, SPSS, IBM SPSS, Madrid, España). RT-qPCR and dual luciferase reporter assay were performed in triplicate. Data are presented as the mean ± SEM. Pairwise statistical differences between groups were determined using Student's *t*-test. *p* < 0.05 was considered to indicate a statistically significant difference.

Statistical analysis and visualization of clinically relevant data and sequencing results are all implemented in R (version: 3.6.3). The R packages that we used are as follows: volcano plots, correlation analysis plots, forest plots, and risk score plots: ggplot2 package; heat map: ComplexHeatmap package; GO/KEGG enrich analysis, clusterProfiler package [8], http://org.Hs.eg.db package, and GOplot package; Kaplan-Meier analysis: survminer package and survival package; time-dependent ROC curve and timeROC package; calibration plot, nomogram graphs, and rms package; decision curve analysis (DCA), survival package, and stdca.R (https://www.mskcc.org/departm-ents/epidemiology-biostatistics/biostatistics/decision-curve-analysis).

## 3. Results

### 3.1. Protein and mRNA Expression Levels of RALA Are Upregulated in OS

**Figure 1:**
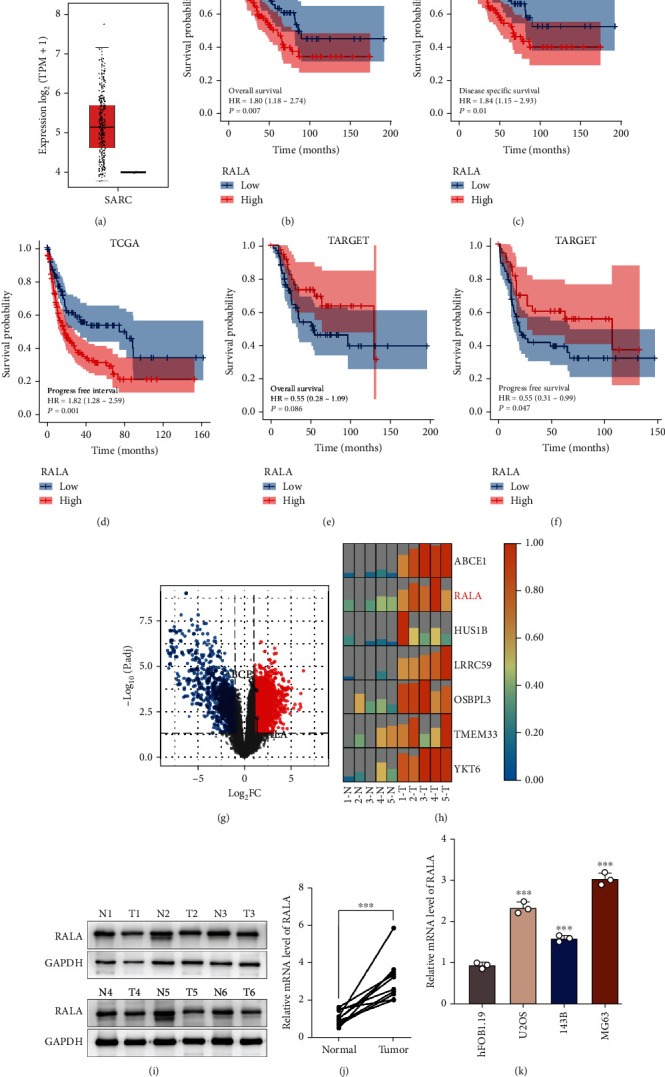
Protein and mRNA expression levels of RALA in OS. (a) mRNA expression level of RALA in OS was analyzed using GEPIA2. (b–f) Survival probability of OS patients in high- and low-RALA groups in TCGA and TARGET database using Kaplan-Meier analysis. (b–d) Overall survival, disease-specific survival, and progress-free survival in TCGA. (e, f) Overall survival and disease-free survival in TARGET. (g, h) RNA-sequencing analyses showed differentially expressed genes, using volcano plots (g) and heat map (h). (i, j) RALA expression status was detected in fresh OS tissues and paired normal specimens. Protein expression level was detected using Western blotting. (i) mRNA expression level was detected using RT-qPCR. ^∗∗∗∗^*p* < 0.0001. mRNA expression level in osteoblast (HFOB1.19) and OS cell lines (U2OS, 143B, and MG63) was detected using RT-qPCR. ^∗∗∗^*p* < 0.001.

Because of the low incidence of osteosarcoma and lack of samples in online databases, we ran the bioinformatic analysis by using datasets of sarcoma including osteosarcoma. Compared with the normal tissues from GTEx, sarcoma tissues from TCGA showed a higher mRNA expression level of RALA (Figure 1(a)). In addition, the high mRNA expression level of RALA conferred significantly shorter survival in terms of overall survival, progress-free survival, and disease-specific survival in these patients (Figures 1(a)–1(c)). At the same time, we explored the relationship between RALA mRNA expression level and overall survival, progress-free survival, and disease-specific survival in the TARGET database using Kaplan-Meier analysis (Figures 1(e) and 1(f)). The results were consistent with TCGA, showing that a higher RALA mRNA expression level was correlated with a shorter survival duration. These data suggest that the amplification of RALA may affect the biological process of OS. The same difference was observed in our RNA-sequencing results (5 OS tissues vs. 5 normal tissues) using volcano plot. MRNA expression levels of RALA in each tissue are shown in a heat map (Figures 1(g) and 1(h)). In addition, we detected the expression level of RALA in 6 pairs of fresh OS tissues and normal tissues and found that RALA protein and mRNA expression levels in the tumor specimens were higher than those in the paired normal specimens (Figures 1(i) and 1(j)). RT-qPCR results showed that the mRNA expression level of RALA was increased in OS cell lines (U2OS, 143B, and MG63) as compared with the osteoblast cell line HFOB1.19 (Figure 1(k)). Among them, MG63 exhibited the highest expression level of RALA and therefore was used for performing the subsequent experiments. In summary, upregulation of RALA was widespread in OS and may be associated with the development and progression of OS, leading to a worse prognosis.

### 3.2. Molecular Interaction Network of RALA in OS

**Figure 2:**
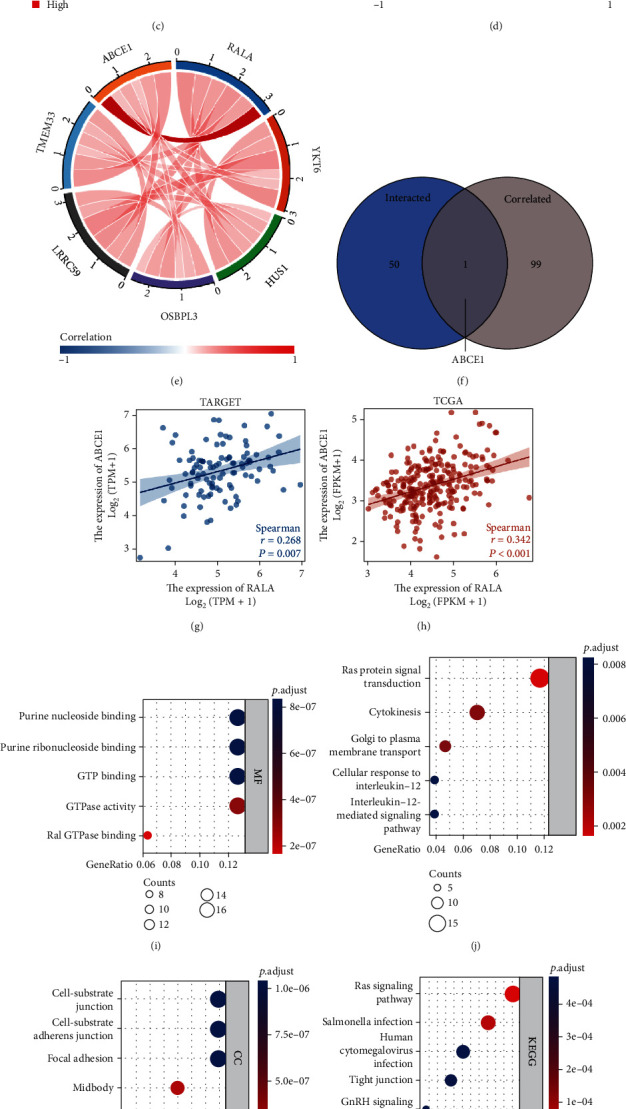
Molecular network and function of RALA in OS. (a) Molecular interaction network of RALA was analyzed using the STRING tool. (b–e) TOP 5 coexpression genes in different tumors were downloaded from GEPIA2. Correlation between the 5 genes; RALA and ABCE1 expression were presented in sarcoma tissues of TCGA using coexpression heat map (b) and chord diagram (d). Data of OS in TARGET was presented in (c) and (e). ^∗^*p* < 0.05; ^∗∗^*p* < 0.01; ^∗∗∗^*p* < 0.001. (f) An intersection analysis of the SND1-binding and correlated genes was conducted. (g, h) Coexpression degree between RALA and ABCE1 in TCGA (g) and TARGET (h) dataset. (i–m) KEGG/GO enrich analyses were performed based on the 50 RALA-binding and 100 interacted genes, containing MF (i), BP (j), CC (k), KEGG pathway (l), and KEGG/GO network (m). (n–s) Functional experiments were performed in MG63 cells. Proliferative ability was detected using EDU assay with representative images (n) and quantitative analysis (q); nuclei stained with DAPI (blue); proliferating cells stained with EDU (red). Migratory ability was detected using wound healing assay with representative images (o) and quantitative analysis (p). Invasive ability was detected using invasion assay with representative images (s) and quantitative analysis (r); cells that pass through Matrigel are stained with 0.2% purple crystal (purple). ^∗∗^*p* < 0.01.

A total of 50 RALA-binding proteins that had been proved in previous experiments were presented using STRING tool (Figure 2(a)). The GEPIA2 (http://gepia2.cancer-pku.cn/) tool was used to combine all tumor expression data of TCGA, from which the top 100 genes that correlated with RALA expression were obtained. Among them, six genes (HUS1, LRRC59, OSBPL3, TMEM33, YKT6, and ABCE) exhibited the highest expression level, and details are shown in the RALA coexpression heat map in Figure 2(b). The correlation between these genes is shown in a chord diagram (Figure 2(d)). The relevance of these results was verified in the TARGET database, and the same phenomena were observed (Figures 2(c)–2(e)). Scatter plots of the associations of the six genes (HUS1, LRRC59, OSBPL3, TMEM33, YKT6, and ABCE) with RALA in TCGA dataset and TARGET dataset are presented in Figure S2 A-B and Figures 2(h) and 2(g). However, intersection of the genes of the two datasets from TCGA and STRING showed that ABCE1 was shared by both as shown in the Venn diagram (Figure 2(f)). The correlation degree (*R* = 0.450, *p* = 0.007) between RALA and ABCE1 is shown in Figure 2(h), while its *R* value was 0.342 (*p* < 0.001) in TARGET dataset (Figure 2(g)). Not surprisingly, ABCE1 was highly expressed in OS group in our RNA-sequencing results (Figures 1(g) and 1(h)). The abnormal ABCE1 mRNA expression level and correlation with RALA are shown in the coexpression heatmaps and chord maps (Figures 2(b)–2(e)). Significant differences in survival were also observed between the high- and low-ABCE1 groups by processing the patient clinical information and RNA-sequencing results from the TCGA and TARGET dataset (Figure S1C-G). This means that research on ABCE1 is helpful to uncover the key role of RALA in OS. RALA upregulation in a concerted manner with ABCE1 was significantly associated with worse outcomes of OS patients. A simultaneous action on ABCE1 and RALA may offer a novel therapeutic strategy for OS.

### 3.3. RALA Promotes OS Progression In Vitro

To gain further insights into the functional impact of RALA in OS, we performed KEGG and GO enrichment analyses. KEGG analysis showed that RALA might be involved in the pathogenesis of OS via the “Ras signaling pathway” (Figure 2(l)). GO enrichment analysis suggested that the main molecular function of RALA in OS may be related to the “GTPase activity” and “Ral GTPase binding,” and the main role of RALA in the biological process may be related to “Ras protein signal transduction,” “cytokinesis,” and “Golgi to plasma membrane transport” (Figures 2(i) and 2(j)). RALA was mainly distributed in the midbody and flemming body in OS (Figure 2(k)). The combined results of KEGG and GO enrichment analyses are shown in a network diagram (Figure 2(m)).

To study the effect of RALA on OS, a mammalian expression plasmid encoding the human RALA ORF and an empty plasmid were constructed. Western blotting and RT-qPCR showed that the protein and mRNA expression levels of RALA in MG63 cells transfected with the overexpression plasmid were higher than those in MG63 cells transfected with empty plasmid (Figure S1H-I), which proved the efficacy of the overexpression plasmid. The effect of RALA on the proliferative ability of OS was investigated with EdU assay. The results showed that MG63 cells overexpressing RALA had a higher proportion of EdU-positive cells (Figures 2(n)–2(q)). Wound healing assay showed that the healing distance was significantly longer in MG63 cells transfected with RALA overexpression plasmids (Figures 2(o) and 2(p)). Additionally, invasion assay showed that RALA promoted more MG63 cells (stained with purple crystal) crawling through the Matrigel to the lower surface of the chambers (Figures 2(r) and 2(s)). All these functional experiments demonstrated that RALA could promote the proliferation, migration, and invasion of OS.

### 3.4. Immune Infiltration Analysis and DNA Methylation Analysis of RALA in OS

**Figure 3:**
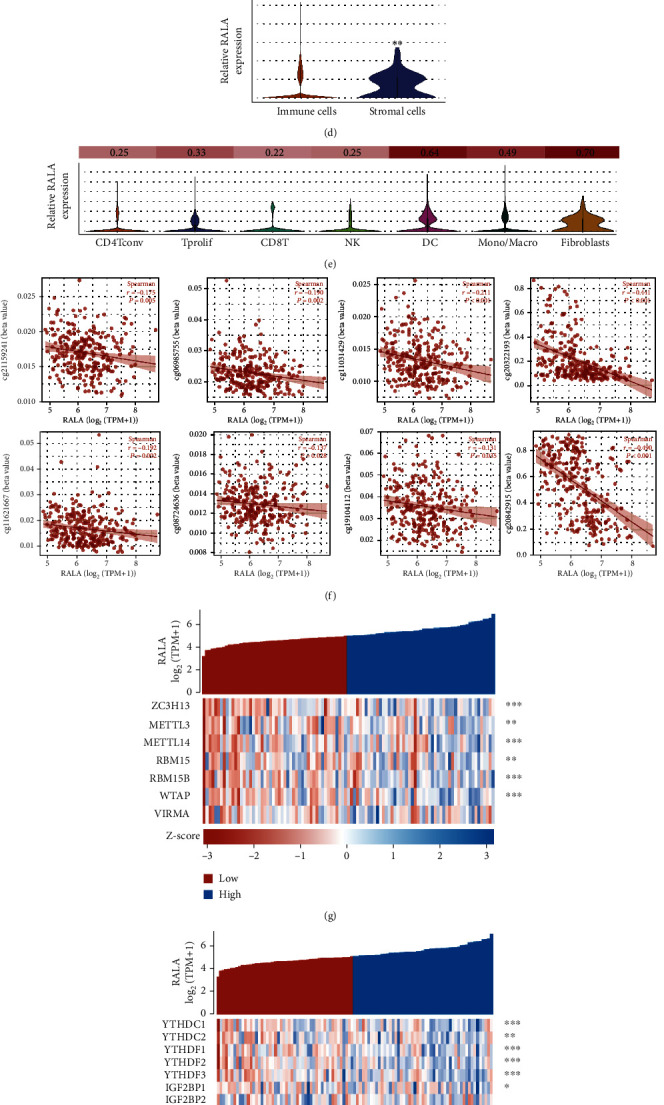
Immune infiltration analysis and DNA methylation analysis of RALA in OS. (a, b) Enrichment scores (a) of RALA in different immune cells in sarcoma. The positive correlation and significance were given in the heat map (b). ^∗∗^*p* < 0.01; ^∗∗∗^*p* < 0.001. (c) Correlation between RALA and immune cells was presented using TIMER tool. (d, e) Single-cell sequencing results show differential expression of RALA in immune cells and stromal cells (d). Expression levels of RALA in different immune cells were given in (e). ^∗∗^*p* < 0.01. (f) Correlation between RALA expression and DNA methylation level was detected in different sites of RALA TSS in OS. (g–j) Correlation between m6A-related genes and RALA in OS tissues from TARGET was presented using coexpression heat map, containing writers (g), reader (h), and erasers (i). Positive correlation and significance were given in the heat map (j). ^∗^*p* < 0.05; ^∗∗^*p* < 0.01; ^∗∗∗^*p* < 0.001.

The tumor microenvironment (TME) theory has been acknowledged by more researchers, and immune infiltration is regarded as the most important part of TME. Previous studies found that RALA involved in immune response of NK cells [11]. To determine whether RALA was involved in the alteration of immune infiltration during OS development and progression, we first compared the enrichment scores of immune cells in high- and low-RALA groups from TCGA database. The results showed that the RALA expression was decreased in DC, NK cells, NK CD56 bright cells, mast cells, Tgd, and Th17 cells, while it was increased in neutrophils, macrophages, Th1 cells, and Th2 cells (Figure 3(a)). Their correlation, significance, and sample-specific expression are shown in Figure 3(b) and Figure S2A. The association of common immune cells with RALA was also calculated with Timer stool (Figure 3(c)). At the same time, the data downloaded from Timer showed that the cumulative survival of different immune cells was different between high- and low-RALA groups (Figure S2B), suggesting that the regulatory effect of RALA on immune cells affected the occurrence and progression of OS. What is more, the single-cell sequencing data obtained from TISH showed that the mRNA expression level of RALA in immune cells of sarcoma mice was significantly lower than that in stromal cells. RALA expression level and distribution in various immune cells are shown in Figure 3(e) and Figure S2D using violin plots and UMAP. Altered immune entry levels in OS may be associated with abnormal RALA amplification (Figure S2C).

The mechanisms leading to aberrant BATF2 expression remain unclear. DNA methylation played a key factor in regulating gene expression. In eukaryotes, the modification of Cap at the 5′ end and poly A at the 3′ end plays a very important role in transcriptional regulation. Therefore, we analyzed the methylation within 5000 bp upstream and 5000 bp downstream of the RALA transcription start site (TSS) in OS tissues and found a total of 8 sites with negative correlations between methylation level and RALA expression level (Figure 3(f)). Among them, M6A methylation and its association with gene regulation have been described and studied in various human tumors [12]. We examined m6a methylation-related genes and found that the level of m6A methylation writers (Figure 3(g)), readers (Figure 3(h)), and erasers (Figure 3(i)) was strongly correlated with the expression level of RALA. The heat map showed that different classes of m6A-methylated genes were associated with RALA (Figure 3(j) and Figure S2E). These findings imply that there is a high probability that m6A methylation is responsible for the abnormal expression of RALA in OS.

### 3.5. RALA Promotes OS In Vivo

**Figure 4:**
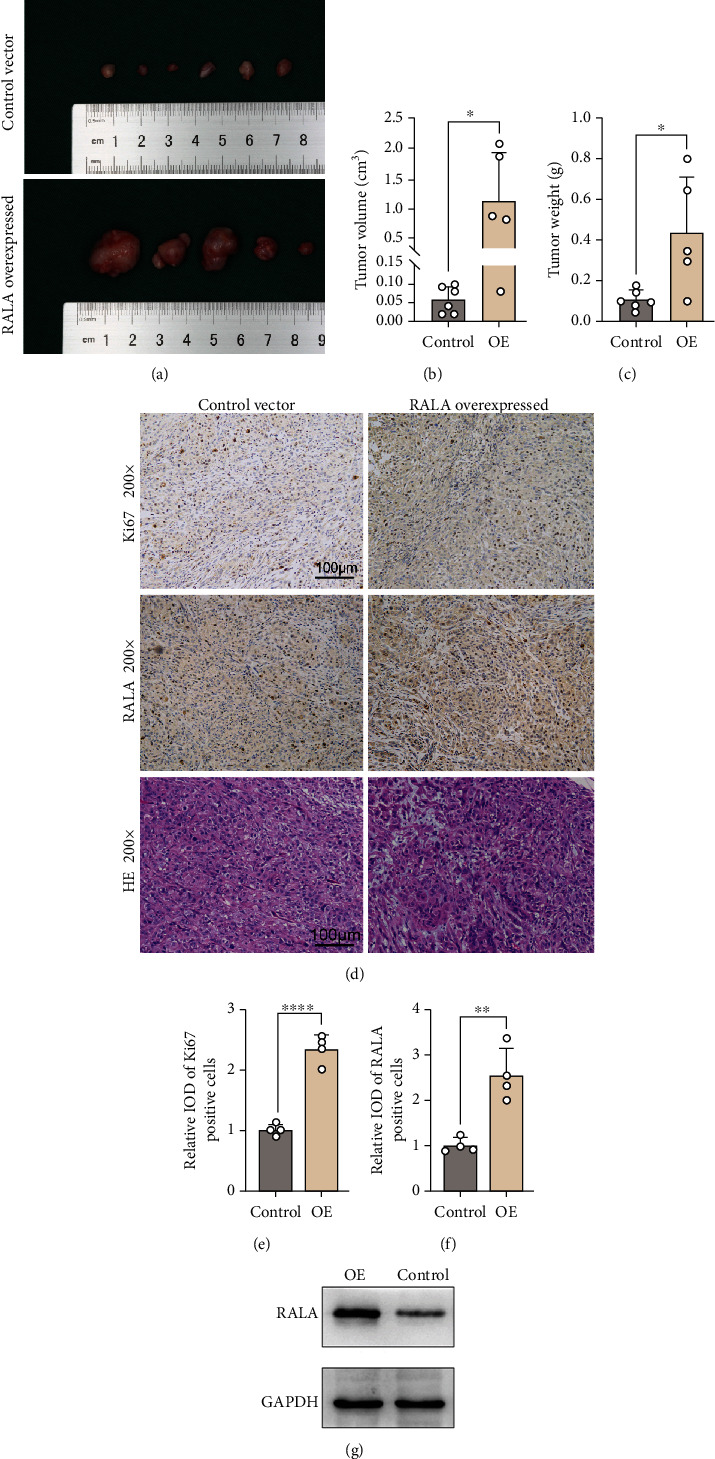
Effects of RALA overexpression on growth of OS xenografts in mice. (a–c) Tumors harvested from male SCID mice (nu/nu; age, 6 weeks; weight, 18 g) were subcutaneously injected into RALA-overexpressed or control MG63 cells (2 × 106 cells/mouse, six mice/group). Photos of tumors (a), tumor volume (b), and tumor weight (c) were presented. ^∗^*p* < 0.05. (d–f) H&E staining (d) was performed on xenografted tumors. Protein expression levels of RALA and Ki67 were detected using immunohistochemical assay (d) and quantitatively analyzed in (e) and (f). ^∗∗^*p* < 0.01; ^∗∗∗∗^*p* < 0.0001. (g) Protein expression levels of RALA in OS xenografted tumors were detected using Western blotting.

The effect of RALA on OS growth was envaulted in vivo using OS xenografts in SCID mice. MG63 cells transfected with RALA overexpression plasmid or control vector were inoculated into the armpit of nude mice, and 24 days later, tumors were evaluated. The result showed that tumors stripped from OS xenografting mice in RALA overexpression group were heavier and larger than those in the control group (Figures 4(a)–4(c)). The maximum single tumor in RALA overexpression group was 2.05 cm^3^ in size and 0.8 g in weight (Figures 4(b) and 4(c)). Then, the xenografted tumors were paraffin embedded and H&E stained for IHC assay. The result showed that there were more mitotic cells in the tumors of RALA overexpression group as compared with the control group (Figure 4(d)). IHC staining demonstrated that RALA and Ki67 expression levels in the tumors of RALA overexpression group were higher than those in the control group (Figures 4(d)–4(f)). Both H&E staining and IHC assay demonstrated that RALA promoted OS growth and development. We also examined the protein expression level of RALA in xenografted tumors using Western blotting (Figure 4(g)), and the tumors in RALA overexpression group showed higher protein level of RALA.

### 3.6. RALA as a Prognostic Indicator for OS

**Figure 5:**
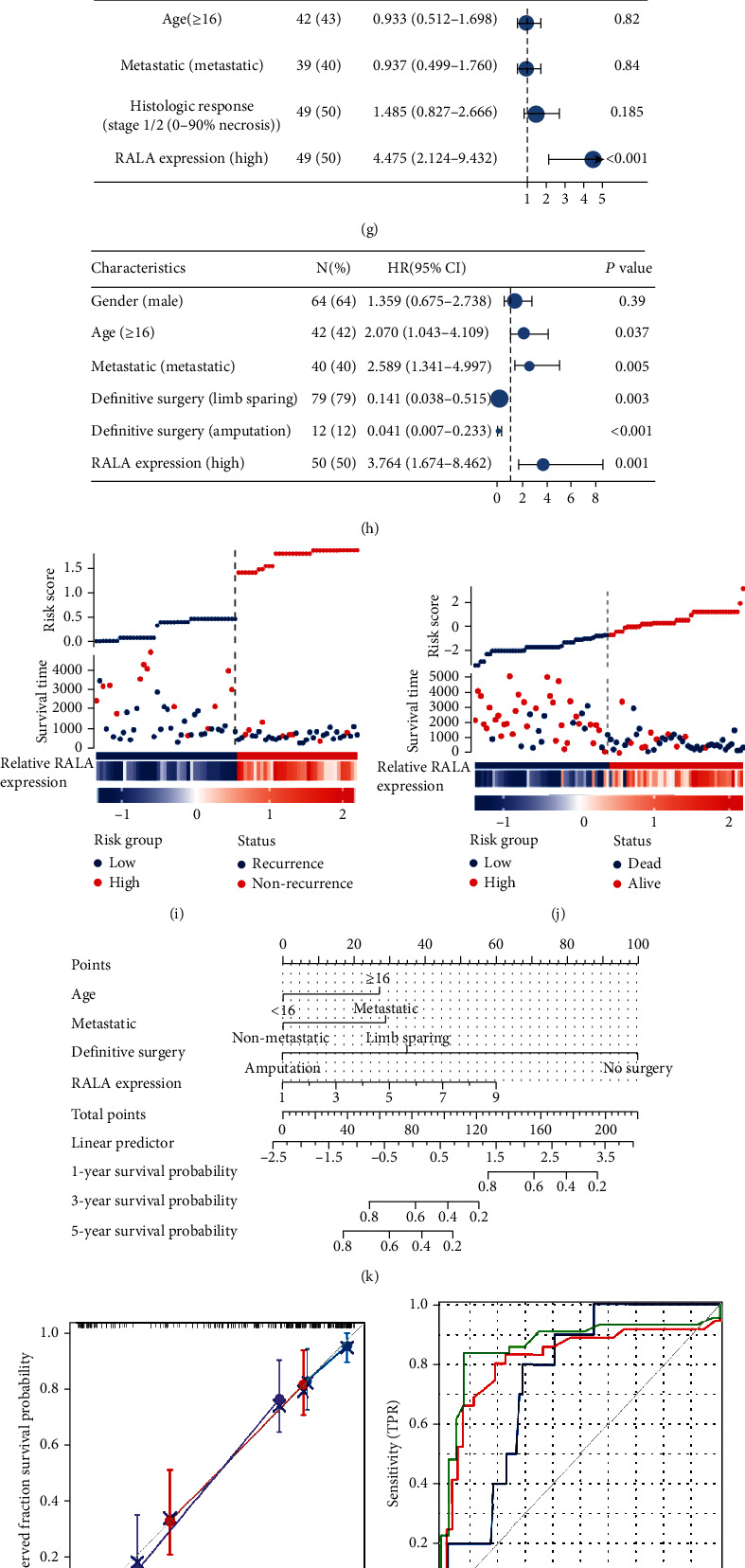
Clinical analyses based on RALA. (a, b) Overall survival (a) and disease-free survival (b) of 100 patients undergoing surgical procedures between 2007 and 2022 were analyzed using Kaplan-Meier analysis. (c–f) Subgroup analyses were performed using Kaplan-Meier analysis. (g, i) Univariate/multivariate Cox proportional hazards regression analyses results of disease-free survival were aggregated using forest plots (g) and risk score plots (i). (h, j) Univariate/multivariate Cox proportional hazards regression analyses results of overall survival were aggregated using forest plots (h) and risk score plots (j). (k–m) Sensitivity and accuracy of nomogram graphs (k) based on RALA were verified by time-dependent ROC curve (l) and calibration model (m). (n–p) 1 (n), 3 (o), and 5 (p) years' benefits from RALA measures in predicting survival outcomes were calculated using DCA curves.

Finally, we explored the possibility of RALA as a prognostic indicator for OS. One hundred OS patients who were admitted in our hospital between 2007 and 2022 were included as research samples to observe the effect of RALA expression on prognosis. According to the relative RALA expression level in their pathological sections, the patients were divided into two groups: RALA high-expression group and RALA low-expression group. There were significant differences in gender (*p* = 0.022), age (*p* < 0.001), metastasis (*p* < 0.001), vital status (*p* < 0.001), disease-free survival (*p* < 0.001), and overall survival (*p* < 0.001) between the two groups (Figure S3A). Difference and confidence interval (CI) in disease-free survival (HR = 2.91, *p* < 0.001) and overall survival (HR = 7.95, *p* < 0.001) between the two groups were shown using Kaplan-Meier analysis (Figures 5(a)–5(b)). Subgroup analysis was performed to compare the effect of RALA expression on overall survival in different subgroups in terms of age (patients < 16 years (HR = 4.03, *p* < 0.001) and ≥16 years (HR = 13.55, *p* < 0.001)) and metastasis (yes (HR = 25.63, *p* = 0.002) and no (HR = 4.70, *p* < 0.001)) (Figures 5(c)–5(g)).

To incorporate RALA expression into the prognostic indicators of OS patients and establish a model to score OS survival probability, univariate/multivariate Cox proportional hazards regression modeling was performed to calculate the relationship between multiple related factors and death/overall survival time or recurrence/disease-free survival time, respectively (Figure S3B-C). Finally, the results were aggregated into two forest plots. Age greater than 16 years (HR = 2.589, 95% CI: 1.341-4.997, *p* = 0.005), metastatic tumors (HR = 2.070, 95% CI: 1.043-4.109, *p* = 0.037), and high expression of RALA (HR = 3.764, 95% CI: 1.674-8.462, *p* = 0.001) all increased the overall risk of survival, while surgical treatment (limb sparing, HR = 0.141, 95% CI: 0.038-0.515, *p* = 0.003; amputation, HR = 0.041, 95% CI: 0.007-0.233, *p* < 0.001) was associated with decreased mortality (Figure 5(h)). However, the result of DFS-related univariate/multivariate Cox proportional hazards regression modeling showed that the probability of recurrence was only related to RALA expression (HR = 4.475, 95% CI: 2.214-9.432, *p* < 0.001), and it was difficult to support a relatively reliable recurrence risk model (Figure 5(g)). We used risk factor association plots to present RALA expression level, overall survival/disease-free time, and outcome and risk score as calculated by multivariate Cox proportional hazards regression modeling for all patients. It was found that a higher RALA expression level was associated with a shorter overall survival time, a worse outcome, and a higher risk score (Figures 5(i) and 5(j)).

To facilitate the clinical use of the RALA-related risk model for survival assessment of OS patients in clinical work, we constructed a nomogram graph to predict 1-, 3-, and 5-year survival probability (Figure 5(k)). Calibration plot at 1, 3, and 5 years indicated moderate calibration of the nomogram (Figure 5(l)). The sensitivity of the model was indicated by the AUC of time-dependent ROC at 1 (AUC = 0.759), 3 (AUC = 0.820), and 5 (AUC = 0.868) years (Figure 5(m)). Finally, the DCA curves were used for evaluating the benefits of incorporating RALA expression into the OS risk assessment model. It was found that the RALA expression model had little benefit but no side effect in assessing the survival of OS patients in the first year (Figure 5(n)), and there was a clear positive benefit in assessment at three years (Figure 5(o)). When the evaluation period was extended to five years, the inclusion of RALA expression in the OS risk assessment model showed significant and considerable benefits (Figure 5(p)). These results concluded that RALA as a marker to construct a prognostic prediction model for OS could provide patients with long-term benefits.

## 4. Discussion

The current first-line treatment for OS is a combination of chemotherapy and surgery, containing neoadjuvant chemotherapy, surgical resection, and postoperative adjuvant chemotherapy. What is even more regrettable is that the existing standard regimens have not substantially improved the survival rate of OS patients in the past three decades. Therefore, it is necessary to find new treatment strategies. Osteosarcoma is a very heterogenous disease entity, and there are multiple factors that have an influence on the prognosis of osteosarcoma. In recent years, the finding of osteosarcoma-associated oncogenes has resulted from the application of the methods of molecular biology. It is more efficient to screen from existing targets that have proven to be effective for the treatment of other tumors. RAS (KRAS, NRAS, and HRAS) is the most frequently mutated gene family in cancers, especially lung, colorectal, and pancreatic cancers [13]. RAS-related pathways such as Ras-PI3K, Ras-ERK1/2, and RAS-MAPK involved in OS progression have also been preliminarily validated [14]. However, for more than three decades, the development of effective therapeutics to inhibit RAS-driven oncogenesis has eluded the field, and RAS was thought to be “undruggable” [15]. Drugs that simply regulate RAS expression have not achieved the desired effect. In this case, RAS-related genes seem to be a promising alternative. Activation of RALA has been shown to be critical for Ras-induced tumorigenesis of human cells [5]. The contribution of RALA in growth of other types of tumors has been established, but it has not been investigated in OS [9]. In this study, we provided a comprehensive bioinformatics and functional analysis of RALA in OS.

We first demonstrated that the mRNA and protein expression levels of RALA in OS were higher than those in normal tissues through various methods including bioinformatics means (TCGA and TARGET), RNA-sequence, Western blotting, and RT-qPCR. Meanwhile, a significant correlation between this differential expression and clinical outcomes was verified in TCGA dataset and TARGET dataset. This means that RALA is a plausible trigger point for our study. Further, in vitro and in vivo assays showed that high RALA expression may promote OS cell proliferation, migration, and invasion. The core part of our experiment is our finding that RALA could be used as a prognostic indicator of OS. In the current mainstream treatment, the American Joint Committee on Cancer (AJCC) AJCC Cancer Staging Manual and The Musculoskeletal Tumor Society (MSTS) staging system are generally used to grade and evaluate the prognosis of OS [16]. However, interventions based on these tumor staging methods have not been effective in classifying OS patients into finer subtypes. Knowing that different expression levels of RALA in OS patients led to different survival duration and outcomes, we established a model for predicting patient prognosis by combining RALA with other clinical indicators, including the information of the patients who underwent surgery in Jinling Hospital in the past 30 years. We first used the univariate/multivariate Cox proportional hazards regression modeling to calculate the risk scores of these patients, then incorporated RALA into the prognostic scoring model as an indicator, and finally verified its sensitivity and accuracy by time-dependent ROC curve and calibration model, respectively. DCA curves also showed that OS patients benefited significantly from RALA measures in predicting survival outcomes after 3 years.

In addition, we observed an interesting phenomenon that the differential expression of RALA not only featured in tumor and normal tissues but was present in immune cells. Based on the enrichment score provided by TIMER tool, we found that the expression of RALA was less expressed in several key immune cells. A single-cell sequencing result of sarcoma directly described the lower expression of RALA in different immune cells [17]. People have recognized that immunity plays an important role in tumor development since more than 10 years ago when they began investigating the immune evasion mechanisms of tumors [18]. The recent emergence of immune checkpoints has changed the treatment of many solid and hematological tumors [19]. However, the clinical response of immune checkpoint inhibitors in recurrent and metastatic OS is not optimistic in that the overall response rate is less than 10%. The response rate of pembrolizumab is 4.5% (SARC028 clinical trial) [20], and the response rate of pembrolizumab combined with metronomic cyclophosphamide in the Pembrosarc clinical trial is 6.7% [21]. Although all these results suggest that targeting a single immune target in OS is not satisfactory, it does not deny the potential of immunotherapy for OS. Unlike other primary tumors, the TME of OS involves the bone marrow, a highly dynamic environment composed of bone cells, immune cells, and stromal and vascular cells, embedded in a mineralized extracellular matrix. In this context, it fits well the idea that the pathophysiology of OS is strictly dependent not only on the molecular events underlining osteoblast differentiation but also on the interaction with the other cell types residing in the BM [22].

The Ral GTPases, directly or indirectly, appear to be implicated in triggering diverse immune signaling pathways [9, 23]. Like other small GTPases, Ral GTPases are molecular switches that can be toggled between inactive GDP-bound and active GTP-bound states to regulate diverse and critical cellular functions. Theoretically, Ral GTPases including RALA probably modulate the proliferation of immune cells and their functions. NK cells are key components of the immune response to virally infected and tumor cells. Recognition of target cells initiates a series of events in NK cells that culminates in target destruction via directed secretion of lytic granules. One previous study suggested that RALA and RALB contribute to the regulation of NK cells. RALA regulated granule polarization toward the immunological synapse and the subsequent process of degranulation. They found that silencing of RALA impaired NK cell cytotoxicity [11]. In our study, we found that RALA expression was decreased in NK cell which was consistent with abovementioned study (Figure 3(a)). Besides this, the cumulative survival analysis indicated that high expression of RALA in immune cells predicts the better prognosis (Figure S2B), suggesting that the regulatory effect of RALA on immune cells affected the occurrence and progression of osteosarcoma. Thus, in the tumor microenvironment of osteosarcoma, the imbalance in RALA expression between osteosarcoma cells and immune cells may be the cause of aggressive tumor progression, which is one of the highlights of this study. The research on the correlation between RAS-related pathways and immune cells is also relatively rare. Our findings focused on the relationship between the RAS-related pathway and immune cells and the relationship between OS and mechanistic cells, which may bring new ideas for finding new targets for OS immunotherapy.

Despite the strengths in this study, there were some limitations. Firstly, one of the distinguishing features of osteosarcoma is the heterogeneity of its gene expression, i.e., the gene expression of osteosarcoma tissues in different patients may be completely different. With the advancement of research methods, the results of single-cell sequencing and spatial transcriptome sequencing have shown that several completely different phenotypes can appear within a solid tumor. It is obviously far from enough to provide key molecules for the targeted therapy and immunotherapy of osteosarcoma only through research of RALA. Patients with osteosarcoma need more individualized treatment. Secondly, the precise molecular mechanisms upstream and downstream of RALA have not yet been fully understood and are yet to be systematically studied. RALA may be regulated by various molecular modalities: pretranscriptionally, posttranscriptionally, and posttranslationally, which deserves the next step study. Although the preliminary results showed that there was a high probability that m6A methylation is responsible for the abnormal expression of RALA in OS, this remains to be tested in trials. Thirdly, we found that expression of the ABCE1 gene was highly correlated to that of RALA, and there was significant difference in survival between the high- and low-ABCE1 groups by processing the patient clinical information and RNA-sequencing results from the TCGA and TARGET dataset. However, gene-gene interactions between ABCE1 and RALA were unclear, and the molecular details of the involved mechanisms are currently lacking. Finally, further clinical trials are needed to validate the prognostic prediction power of the RALA-related risk model for survival assessment of OS patients.

In summary, we explored the identity of RALA in the biological process of OS, its potential therapeutic direction, and the possibility of using it as a prognostic indicator by means of bioinformatics, molecular biology, and clinical statistics. Our study provides a new way of thinking for the discovery of therapeutic targets for OS through the combination of biological prediction and molecular biological verification.

## Data Availability

The raw public data were downloaded from the Cancer Genome Atlas (TCGA, https://portal.gdc.cancer.gov/) dataset, Genotype-Tissue Expression (GTEx, https://gtexportal.org/) dataset, Therapeutically Applicable Research To Generate Effective Treatments (TARGET, https://ocg.cancer.gov/programs/target) dataset, Tumor Immune Single-cell Hub (TISH, http://tisch.comp-genomics.org/), STRING (https://cn.string-db.org/), and the Timer (https://cistrome.shinyapps.io/timer/) dataset. Patient information for OS and sarcoma was downloaded from TARGET and TCGA. Information on normal tissues was downloaded from GTeX. Immunization-related information was downloaded from Timer. Single-cell sequence data were downloaded from TISH. Protein-protein data were downloaded from STRING.

## Abbreviations

OS: Osteosarcoma
Ral GTPases: Ras-like guanosine triphosphatases
RALA: RAS-like proto-oncogene A
GAPDH: Glyceraldehyde-3-phosphate dehydrogenase
UTR: Untranslated region
miRNA: mRNA: messenger RNA
EdU: 5-ethynyl-2′-deoxyuridine
Ki67: Antigen KI67
BM: Bone marrow.

## Supplementary Materials

Supplementary 1 Figure S1: analysis of the RALA-related gene expression status and outcome based on ABCE1 and validity verification of RALA overexpression plasmid, related to Figure 2. (a, b) Correlation between HUS1, LRRC59, OSBPL3, TMEM33, YKT6, and RALA in TCGA (a) and TARGET (b). (c–g) Survival probability of osteosarcoma patients in high- and low-ABCE1 groups in TCGA and TARGET database using Kaplan-Meier analysis. (c–e) Overall survival, disease-specific survival, and progress-free survival in TCGA. (f, g) Overall survival and disease-free survival in TARGET. (h, i) RALA expression status was detected in MG63 cells that transfected with RALA overexpression plasmid. mRNA expression level was detected using RT-qPCR. (h) Protein expression level was detected using Western blotting. (i) ^∗∗∗^*p* < 0.001; ^∗∗∗∗^*p* < 0.0001.

Supplementary 2 Figure S2: enrichment and cumulative survival based on RALA and immune cells, single-cell sequencing results, and correlation between m6A-related genes and RALA, related to Figure 3. (a) Correlation between enrichment of different immune cells and RALA expression. (b) Cumulative survival of different immune cells in high- and low-RALA groups were presented using TIMER stool. (c) DNA copy events of RALA were presented in different immune cells using TIMER stool. (d) RALA expression level and distribution in various immune cells were shown using UMAP from TISH. (e) Correlation between different classes of m6A methylated genes and RALA in tissues from TARGET database.

Supplementary 3 Figure S3: baseline data and univariate/multivariate Cox proportional hazards regression modeling, related to Figure 5. (a) Baseline data of 100 OS patients undergoing surgical procedures between 2007 and 2022. (b) Disease-free survival of osteosarcoma patients was analyzed using univariate/multivariate Cox proportional hazards regression modeling. (c) Overall survival of osteosarcoma patients was analyzed using univariate/multivariate Cox proportional hazards regression modeling.
