# Supplementary figures and images for: Identification of RALA as a Therapeutic Target and Prognostic Predictor of Osteosarcoma
Source: Biomed Res Int. 2023 Feb 7;2023:1150768. doi: 10.1155/2023/1150768 (PMC9936457; doi:10.1155/2023/1150768)

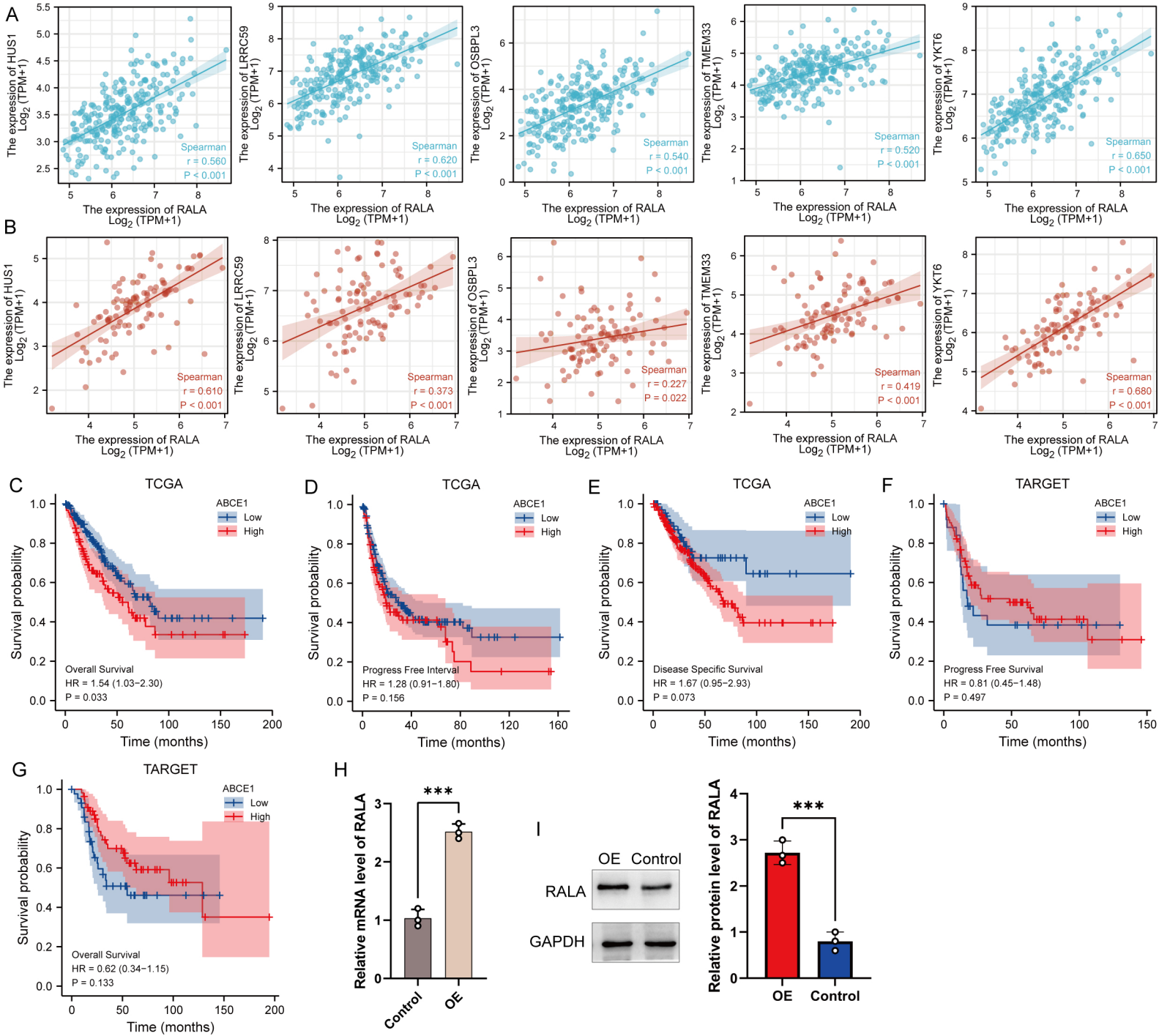

Supplement: Supplementary 1 — Figure S1: analysis of the RALA-related gene expression status and outcome based on ABCE1 and validity verification of RALA overexpression plasmid, related to Figure 2. (a, b) Correlation between HUS1, LRRC59, OSBPL3, TMEM33, YKT6, and RALA in TCGA (a) and TARGET (b). (c–g) Survival probability of osteosarcoma patients in high- and low-ABCE1 groups in TCGA and TARGET database using Kaplan-Meier analysis. (c–e) Overall survival, disease-specific survival, and progress-free survival in TCGA. (f, g) Overall survival and disease-free survival in TARGET. (h, i) RALA expression status was detected in MG63 cells that transfected with RALA overexpression plasmid. mRNA expression level was detected using RT-qPCR. (h) Protein expression level was detected using Western blotting. (i) ∗∗∗p < 0.001; ∗∗∗∗p < 0.0001. [file 1150768.f1.pdf]

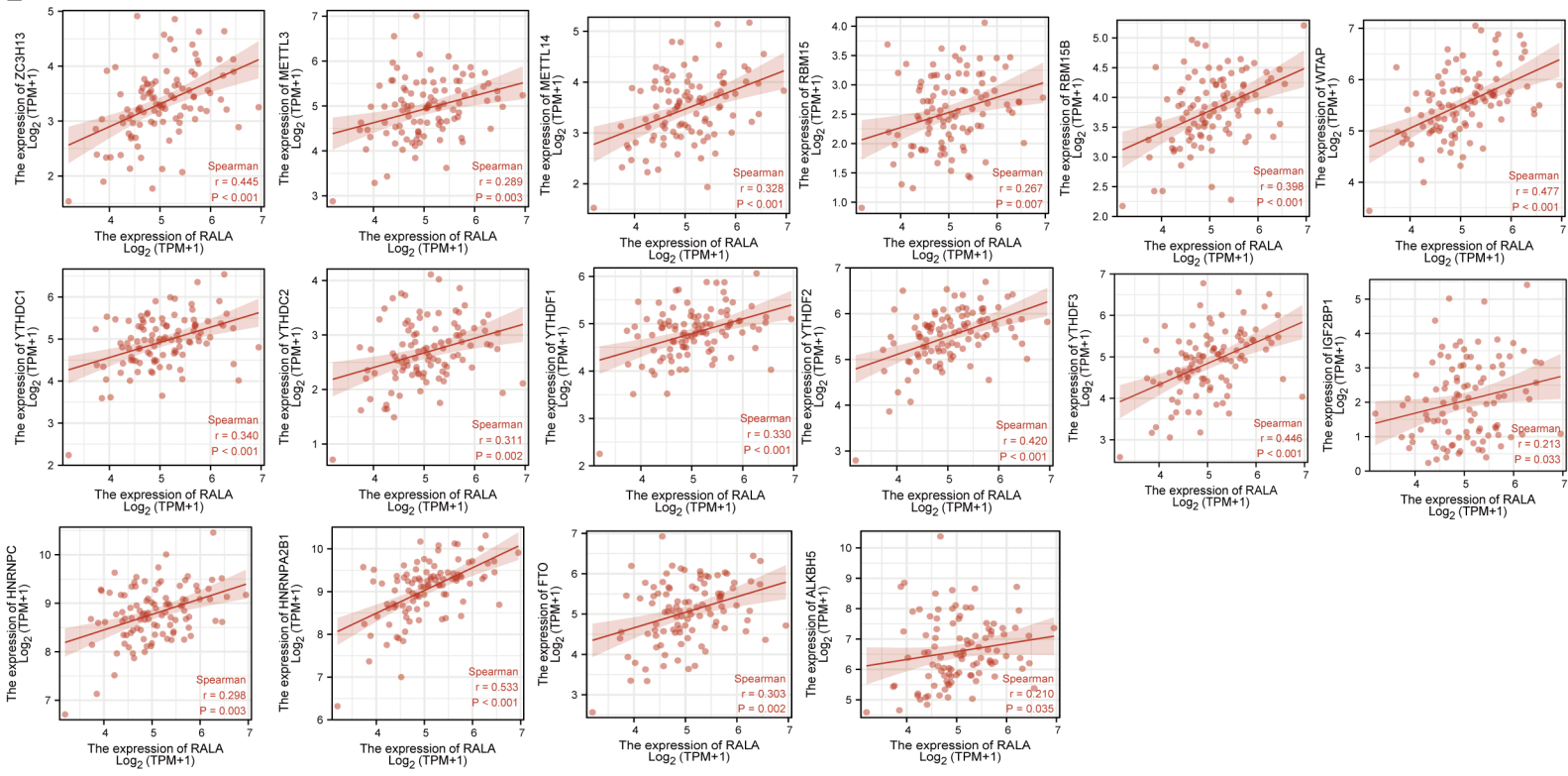

Supplement: Supplementary 2 — Figure S2: enrichment and cumulative survival based on RALA and immune cells, single-cell sequencing results, and correlation between m6A-related genes and RALA, related to Figure 3. (a) Correlation between enrichment of different immune cells and RALA expression. (b) Cumulative survival of different immune cells in high- and low-RALA groups were presented using TIMER stool. (c) DNA copy events of RALA were presented in different immune cells using TIMER stool. (d) RALA expression level and distribution in various immune cells were shown using UMAP from TISH. (e) Correlation between different classes of m6A methylated genes and RALA in tissues from TARGET database. [file 1150768.f2.pdf]
