# Supplementary material for: Identification of RALA as a Therapeutic Target and Prognostic Predictor of Osteosarcoma
Source: Biomed Res Int. 2023 Feb 7;2023:1150768. doi: 10.1155/2023/1150768 (PMC9936457; doi:10.1155/2023/1150768)
Supplement: Supplementary 3 — Figure S3: baseline data and univariate/multivariate Cox proportional hazards regression modeling, related to Figure 5. (a) Baseline data of 100 OS patients undergoing surgical procedures between 2007 and 2022. (b) Disease-free survival of osteosarcoma patients was analyzed using univariate/multivariate Cox proportional hazards regression modeling. (c) Overall survival of osteosarcoma patients was analyzed using univariate/multivariate Cox proportional hazards regression modeling. [file 1150768.f3.pdf]

A

Characteristic	RALA_high_expression	RALA_low_expression	p
n	50	50	
Gender, n (%)			0.022
Female	12 (12%)	24 (24%)	
Male	38 (38%)	26 (26%)	
Age, n (%)			< 0.001
＜16	17 (17%)	41 (41%)	
≥16	33 (33%)	9 (9%)	
Metastatic, n (%)			< 0.001
metastatic	30 (30%)	10 (10%)	
non-metastatic	20 (20%)	40 (40%)	
Histologic response, n (%)			0.421
Stage 1/2 (0-90 % necrosis)	25 (31.6%)	25 (31.6%)	
Stage 3/4 (91-100 % necrosis)	11 (13.9%)	18 (22.8%)	
Definitive Surgery, n (%)			0.651
Amputation	5 (5.3%)	7 (7.4%)	
Limb sparing	43 (45.7%)	36 (38.3%)	
No surgery	2 (2.1%)	1 (1.1%)	
Vital Status, n (%)			< 0.001
Alive	13 (13%)	37 (37%)	
Dead	36 (36%)	13 (13%)	
Unknown	1 (1%)	0 (0%)	
Disease Free Survival Time in Days	365 (225, 576)	1263 (598, 2501)	< 0.001
Overall Survival Time in Days	602 (368, 1022.5)	2108 (1559.75, 3114)	< 0.001

B

Characteristics	Total(N)	Univariate analysis		Multivariate analysis	
		Hazard ratio (95% CI)	P value	Hazard ratio (95% CI)	P value
Gender	98				
Female	36	Reference			
Male	62	1.515 (0.919-2.498)	0.104		
Age	98				
＜16	56	Reference			
≥16	42	1.589 (0.979-2.579)	0.061	0.933 (0.512-1.698)	0.820
Metastatic	98				
non-metastatic	59	Reference			
metastatic	39	1.758 (1.067-2.897)	**0.027**	0.937 (0.499-1.760)	0.840
Histologic response	78				
Stage 3/4 (91-100 % necrosis)	29	Reference			
Stage 1/2 (0-90 % necrosis)	49	1.620 (0.915-2.870)	0.098	1.485 (0.827-2.666)	0.185
Definitive Surgery	92				
No surgery	3	Reference			
Limb sparing	78	0.569 (0.177-1.832)	0.345		
Amputation	11	0.600 (0.154-2.332)	0.461		
RALA expression	98				
low	49	Reference			
high	49	2.910 (1.748-4.844)	**<0.001**	4.475 (2.124-9.432)	**<0.001**

C

Characteristics	Total(N)	Univariate analysis		Multivariate analysis	
		Hazard ratio (95% CI)	P value	Hazard ratio (95% CI)	P value
Gender	100				
Female	36	Reference			
Male	64	1.973 (1.028-3.789)	**0.041**	1.359 (0.675-2.738)	0.390
Age	100				
＜16	58	Reference			
≥16	42	3.900 (2.159-7.046)	**<0.001**	2.070 (1.043-4.109)	**0.037**
Metastatic	100				
non-metastatic	60	Reference			
metastatic	40	2.961 (1.670-5.247)	**<0.001**	2.589 (1.341-4.997)	**0.005**
Histologic response	79				
Stage 3/4 (91-100 % necrosis)	29	Reference			
Stage 1/2 (0-90 % necrosis)	50	1.666 (0.826-3.361)	0.154		
Definitive Surgery	94				
No surgery	3	Reference			
Limb sparing	79	0.140 (0.041-0.481)	**0.002**	0.141 (0.038-0.515)	**0.003**
Amputation	12	0.044 (0.008-0.232)	**<0.001**	0.041 (0.007-0.233)	**<0.001**
RALA expression	100				
low	50	Reference			
high	50	7.946 (4.027-15.681)	**<0.001**	3.764 (1.674-8.462)	**0.001**
